# “I sold my towel and shoes to pay the traditional healer”: Care-seeking costs and productivity losses among snakebite victims in Eastern Province, Rwanda

**DOI:** 10.1371/journal.pntd.0011768

**Published:** 2023-11-20

**Authors:** Janna M. Schurer, Mahlet Tadesse Admasu, Mihigo Bonaventure, Dieudonne Hakizimana, Elijah Murara, Lauren E. MacDonald, Ellen Rafferty

**Affiliations:** 1 Center for One Health, University of Global Health Equity, Butaro, Rwanda; 2 Department of Infectious Disease and Global Health, Cummings School of Veterinary Medicine at Tufts University, North Grafton, Massachusetts, United States of America; 3 University of Washington, Department of Global Health, Seattle, Washington state, United States of America; 4 World Health Organization Regional Office for Europe, Copenhagen, Denmark; 5 Department of Medicine, Faculty of Medicine and Dentistry, University of Alberta, Edmonton, Canada; Fundação de Medicina Tropical Doutor Heitor Vieira Dourado, BRAZIL

## Abstract

Snakebite envenomation (SBE) is endemic to sub-Saharan Africa and generally over-represented in rural, remote, and impoverished agricultural communities. While poverty is an established risk factor, little research has been done to investigate the economic consequences of SBE. This cross-sectional, quantitative study aimed to measure out-of-pocket spending and lost income when a household member was bitten by a snake. In 2020, 732 snakebite survivors from Eastern Province (Rwanda) agreed to complete a survey administered by telephone. The survey focused on participant demographics, income, direct medical and non-medical costs, care-seeking decisions, and lost work during convalescence. Our results suggested that patients incurred the highest mean expenses when they sought care from hospitals (11 307 RWF or 12 USD) or traditional healers (5 836 RWF or 6 USD) but that the highest maximum cost was incurred from traditional healers (300 000 RWF or 313 USD). Across all victims, the total amount paid to traditional healers (3.4 million RWF or 3 537 USD) was 4.7 times higher than all other care providers combined. On average, families lost 111 814 RWF (117 USD) per snakebite in direct treatment costs and indirect productivity losses. Many victims sought care from traditional healers despite being eligible for free medical care. Altogether, this study serves as a reminder of the serious physical and financial consequences associated with SBE and provides justification for new investments into SBE prevention and care.

## Author summary

Snakebite is a life-threatening situation in sub-Saharan Africa, especially for rural and poor populations. Despite being preventable and treatable, it receives little attention from policymakers. The goal of this study was to estimate the financial losses experienced by snakebite victims due to treatment costs and lost work. Interviews with 732 snakebite victims from Eastern Province, Rwanda, indicated that a typical adult lost 12.0% of their annual income due to a single bite. Moreover, caregivers lost time and income when caring for a sick family member. Patients preferentially sought care from traditional healers rather than Community Health Workers, nurses or physicians, even when they qualified for free hospital care. Consequently, victims in our study group paid five times more to traditional healers (3.4 million RWF or 3 537 USD) than to all other care providers combined. Altogether, our analysis suggests that Rwandese lose 7.4 million USD each year in treatment costs and lost work due to snakebite. These results serve as evidence to policymakers that investments to prevention and treatment programs will not only reduce pain and suffering, but also contribute to national goals targeting poverty reduction.

## Introduction

Worldwide, 1.8–2.7 million people are envenomed by snakes annually, resulting in up to 138 000 deaths and 400 000 permanent disabilities [1,2]. Snakebite envenomation (SBE) most frequently affects impoverished populations engaged in subsistence farming and resource extraction. Those at particular risk for mortality include children, due to their small size, and those bitten in rural and remote areas, due to delays accessing appropriate medical treatment [3,4]. Victims who survive envenomation can face long-term physical disability (e.g., blindness, limb amputation), in addition to psychological trauma (e.g., post-traumatic stress disorder) and economic catastrophe. Thus, poverty is both a cause and consequence of SBE [3].

The health burden of SBE in Sub-Saharan Africa (SSA) is estimated to be 1.03 million Disability Adjusted Life-Years (95% CI: 0.8–1.28 million DALYs) annually [5]. Costs associated with SBE can include transport to obtain care, treatment at health facilities (diagnostic tests, physician fees, pharmaceuticals), traditional medicine, patient and caregiver loss of productivity during convalescence, and long-term disability affecting life-long earnings. Families can also experience loss when domestic animals reared for income generation are envenomed [6]. While anecdotal evidence suggests that SBE can be financially catastrophic for those at highest risk, almost no evaluations of treatment costs or productivity losses have been reported in SSA. Existing costing analyses focus primarily on hospital costs [7,8].

The low global investments in prevention, health provider education, and biomedical innovation compared to the high burden of disease make SBE one of the most neglected tropical diseases (NTDs) in the world [1]. In Rwanda, very little is known about the epidemiology, geographic distribution, or burden of SBE. In Eastern Province, the incidence in 2020 was estimated to be 4.9 cases per 1 000 people annually [9]. A previous assessment of hospital records suggested that Eastern Province hospitals admitted more snakebite cases than other hospitals [10]. Those bitten seek care predominantly from traditional healers, sometimes in combination with formal health providers [11]. One reason for this is the common perception that hospital care is unaffordable; however, there have been no studies comparing costs between formal medical care and traditional medicine. The aims of this study were to estimate out-of-pocket costs and household productivity losses among families affected by SBE in Eastern Province, Rwanda.

## Methods

### Ethics statement

This study was reviewed and approved by the University of Global Health Equity Institutional Review Board (UGHE-IRB/2020/012). Verbal informed consent was obtained from all adult participants prior to data collection. In the case of children, informed consent was requested from their parent or guardian.

### Study setting

According to the latest population and housing census conducted in 2022, approximately 13.2 million people call Rwanda home, with most earning income through cash crop or livestock production [12,13]. The population is distributed across four provinces (Eastern, Southern, Northern, Western) and Kigali City and divided into four socioeconomic (ubudehe) categories differentiated according to monthly income and ownership of assets. Ubudehe 1 represents the poorest. Thirteen medically important snakes are also present in Rwanda; these include vipers (*Bitis* spp.), mambas (*Dendroaspis* spp.), cobras (*Naja* spp.), and asps (*Atractaspis* spp.)[14]. The formal health system has a well-organized hierarchy of care starting with Community Health Workers (CHWs) at the village level who refer patients to nurses at health posts/centers, who in turn might refer patients to physicians at district, provincial or referral hospitals [15]. Snake-antivenom (SAV) appropriate against African snakebites is available at 4.2% of health facilities but is not considered affordable according to World Health Organization standards [16]. Only physicians are authorized to administer SAV.

### Study design and tool

In this cross-sectional study, we employed a multiple-choice questionnaire to assess healthcare sought, direct medical and non-medical expenditures, and indirect productivity costs of individuals bitten by snakes in 2020 as well as their caregivers. Topics included participant demographics, personal and household income, direct/indirect costs, and work history. More specifically, the questionnaire included out-of-pocket expenditures at pharmacists, CHWs, traditional healers, health posts/centers, and hospitals as well as roundtrip transportation to reach care and food or lodging while receiving care. The survey did not assess co-pay by health insurance plans. Respondents were able to clarify experiences with open-ended responses when needed. The questionnaire was developed in English, translated to Kinyarwanda, pre-tested, and adapted according to feedback.

### Data collection

This economic analysis was a subset of a larger study designed to obtain a representative estimate of snakebite incidence in Eastern Province, Rwanda. Census data and a cluster sampling approach led to the random selection of three sectors for each of the seven districts to generate the necessary sample size. All villages within target sectors were surveyed. Between 2021 and 2022, our team enlisted CHWs to conduct door-to-door surveys to identify cases of snakebite in 681 villages across six of the seven districts in Eastern Province (Gatsibo, Kayonza, Kirehe, Ngoma, Nyagatare, Rwamagana). A snakebite case was defined as any person who self-reported being bitten by a snake in 2020. Our team was unable to validate whether individuals were envenomed. Paper forms listing the name, age, gender, ubudehe (socioeconomic) status, and phone number of each snakebite victim were filled and returned to Kigali where data were checked for completeness and then manually entered into a MS excel datasheet. In the case where data were incomplete or illegible, forms were returned to CHWs to refill. Between May and November of 2022, seven Kinyarwanda speaking enumerators, trained in the survey tool, telephoned snakebite survivors by telephone, explained the study, and requested verbal participation consent. Enumerators attempted to contact potential participants six times over three consecutive days (2 times per day). Those not responding were classified as not reachable, and their data were not included in the analysis. In the case of missing contact information, the team asked the village CHW to locate the participant, and if necessary, to lend their mobile phone to the prospective participant so that the interview could be conducted. If a snakebite survivor incurred multiple bites in 2020, they were asked to provide answers regarding the first event. Parents or guardians were interviewed when a victim was less than 18 years of age at the time of the interview. Interviews lasted between 30 to 40 minutes and responses were entered directly into Qualtrics, an online survey platform, by enumerators. As snakebite victims were anticipated to be primarily subsistence farmers, enumerators were trained to probe respondents to provide the cash value of income as well as agricultural commodities produced on their farms. For example, a respondent unsure about their earnings might be asked which crops they grew, how many 50 kg sacks of each crop they produced during harvest, and the market rate at that time. Regarding productivity costs, enumerators were instructed to request the number of workdays missed as a direct result of recovering from or caregiving for the acute snakebite event. One member of the study team (MT) monitored incoming data daily to check for errors or inconsistencies. When an error was detected, the enumerator called the respondent back immediately and the database was updated with the correct response.

### Statistical analysis

The final dataset was downloaded from Qualtrics, cleaned, coded, and analyzed using IBM Statistical Package for the Social Sciences v.29. Categorical data (e.g., age, education, health insurance, payment methods) were analyzed descriptively using counts and proportions disaggregated by sex. Continuous data (e.g., income, medical costs, non-medical costs, lost workdays) were summarized by means, minimums and maximums. Mean out-of-pocket direct costs were calculated by summing direct medical and non-medical costs incurred by each victim, then summing the costs across all victims and dividing by the total number of victims. Indirect costs (i.e., productivity losses) for acute care and recovery were calculated by summing the number of workdays lost by patients and caregivers for each snakebite case and then multiplying by the average daily wage in Rwanda. The daily wage was estimated by converting the 2020 World Bank estimate for the Rwanda Gross National Income (GNI) per capita (760 USD/person [17]) to Rwandan Francs (RWF) using the mean 2020 exchange rate (957 RWF = 1 USD [18]) and then dividing by 365 days to obtain a daily wage of 1 993 RWF (2 USD). Total mean household losses were calculated by summing direct and indirect costs for each household, summing across all households, and then dividing the sum by the total number of households with at least one snakebite. Finally, a crude estimate of national out-of-pocket and productivity losses was calculated by multiplying 2020 Eastern Province losses by the provincial incidence rate.

## Results

In total, we confirmed 737 snakebite cases in target communities across the six study districts. Of these, 732 snakebite survivors consented to participate in the full interview, 1 consented but then dropped out, and 4 could not be contacted. Participants were predominantly female (63.4%), lacked formal education (primary or less; 88.7%), and primarily engaged in agricultural production (81.6%; Table 1). The average household had five members (min-max: 1–12), with an average of two income earners (min-max: 0–7) and an average of three dependents (min-max: 0–10). Most (91.0%) had medical insurance, with annual insurance expenditures ranging from zero to 84 000 RWF (88 USD). Among the 666 insured households, 115 belonged to Ubudehe 1 (the poorest category) and incurred no insurance costs, two were covered by a charitable organization, 547 paid the government tariff of 3000 RWF (3 USD) per person, and two paid private insurers. Most victims (84.1%) belonged to the 2^nd^ or 3^rd^ ubudehe categories.

**Table 1:** Demographic characteristics of individuals bitten by snakes across six districts of Eastern Province, Rwanda (N = 732).

Variable	Male (n = 268)	Female (n = 464)	Total (N = 732)
	n (%)		
Age (years)			
≤17	83 (31.0)	96 (20.7)	179 (24.5)
18–34	96 (35.8)	145 (31.3)	241 (32.9)
35–50	62 (23.1)	130 (28.0)	192 (26.2)
>50	27 (10.1)	93 (20.0)	120 (16.4)
Education			
None	59 (22.0)	121 (26.1)	180 (24.6)
Primary	172 (64.2)	297 (64.0)	469 (64.1)
Secondary or higher	37 (13.8)	46 (9.9)	83 (11.3)
Ubudehe category			
1 (poorest)	37 (13.8)	78 (16.8)	115 (15.7)
2	149 (55.4)	254 (54.7)	402 (54.9)
3	82 (30.6)	132 (28.4)	214 (29.2)
4 (wealthiest)	1 (0.4)	0 (0)	1 (0.1)
Occupation (adults)			
Unemployed/retired	15 (8.1)	34 (9.2)	49 (8.9)
Student	3 (1.6)	5 (1.4)	8 (1.4)
Agriculture/labour	147 (79.5)	304 (82.6)	451 (81.6)
Tradesperson	14 (7.6)	16 (4.3)	30 (5.4)
Homemaker	6 (3.2)	6 (1.6)	12 (2.2)
Teacher	0 (0)	3 (0.8)	3 (0.5)
Health insurance			
No	20 (7.5)	46 (9.9)	66 (9.0)
Yes	248 (92.5)	418 (90.2)	666 (91.0)
Snakebite frequency per victim in 2020			
1	255 (95.1)	452 (97.4)	707 (96.6)
2	12 (4.5)	10 (2.2)	22 (3.0)
3	1 (0.4)	1 (0.2)	2 (0.3)
4	0 (0)	1 (0.2)	1 (0.1)
Long-term income changes due to snakebite, among adults reporting income (self-reported)			
Decrease	79 (49.4)	172 (56.2)	251 (53.9)
No change	81 (50.6)	134 (43.8)	215 (46.1)
Increase	0 (0)	0 (0)	0 (0)
Snakebite recovery			
Ongoing disability	3 (1.1)	16 (3.4)	19 (2.6)
Partial recovery	78 (29.1)	141 (30.4)	219 (29.9)
Full recovery	187 (69.8)	307 (66.2)	494 (67.5)

Most respondents were bitten once in 2020, with only 25 (3.4%) bitten two to four times. Snakebite victims sought care most frequently from traditional healers, followed by health posts/centers, hospitals, pharmacists and CHWs (Table 2). They most often travelled by walking or motorbike/bicycle; other transport modes included being carried by family or friends, and being driven in a car or ambulance. Approximately one in ten respondents (12.7%) used no transportation. This was usually because they sought care from a traditional healer who visited them at their home. Average out-of-pocket costs per victim for a single snakebite were highest at hospitals (11 307 RWF), followed by traditional healers (5 836 RWF), pharmacists (3 750 RWF) and health post/centers (1 058 RWF); however, the maximum costs incurred at traditional healers far exceeded all other care providers. Total expenditures across all victims were highest for traditional healers (3.4 million RWF) followed by hospitals (463 575 RWF), health posts/centers (226 155 RWF), and pharmacists (37 400 RWF). No respondents paid CHWs to receive care. Length of stay for hospital inpatients varied from 1 to 45 days (mean = 6 days). No uninsured individuals sought hospital care. Altogether, direct medical and non-medical out-of-pocket expenditures amounted to an average of 7 672 RWF (8 USD) per snakebite. These were paid most often by savings or loans, followed by donations from family/friends and selling property. Some traditional healers allowed individuals to work off the cost of treatment: “I still owe the traditional healer the whole amount, I am planning to go and work/cultivate for him and pay through that.” (ID 159, Female farmer).

**Table 2:** Direct out-of-pocket care seeking costs (2020RWF and 2020USD) by facility type among individuals bitten by a snake in 2020 in Eastern Province, Rwanda.

	Mean, Min—Max (RWF)	Mean, Min—Max (USD)	Total (RWF)	Total (USD)
*Medical* *costs*^*1*^				
Pharmacist (n = 10)	3 750, 0–11 200	4, 0–120	37 400	39
Traditional Healer (n = 561)	5 836, 0–300 000	6, 0–313	3 384 600	3 537
Community Health Worker (n = 6)	0, 0–0	0, 0–0	0	0
Health Post/Center (n = 214)	1 058, 0–60 000	1, 0–63	226 155	236
Hospital (n = 40)	11 307, 0–60 500	12, 0–63	463 575	484
*Non-medical costs* ^*2*^				
Transportation (n = 233)	3 752, 150–25 000	4, 0–26	874 250	914
Hospital food/lodging (n = 34)	15 359, 500–200 000	16, 1–209	629 700	658
**Mean medical costs/patient**	5 617, 0–300 000	6, 0–313		
**Mean non-medical costs/patient**	2 055, 0–212 000	2, 0–222		
**Total mean direct costs/patient**	7 672, 0–300 000	8, 0–313		

^1^Includes all individuals who sought care with provider, including those with no expenses.

^2^Only among those who incurred transportation, food or lodging expenses.

Nearly half (42.8%) of respondents incurred no long-term consequences; the other reported a variety of issues including fear or anxiety related to snakes, food insecurity, interrupted schooling, and/or lasting physical limitations that compromised income generation. Two respondents were pregnant at the time of their bite and suffered miscarriages. Among adult respondents, most (83.7%) earned income prior to the snakebite; of these, 53.9% reported long-term decreased earnings after the bite. According to one respondent, “I am no longer productive as I used to be before the snakebite. I always feel pain in my arm where I was bitten … I cannot cultivate for a long time.” (ID 75, Female farmer). Mean recovery time for victims was 35 days (min-max: 0.5–548) with some stating that they never returned to work or school. Adult victims missed an average of 41 days of work (min-max: 0–365), while students lost 28 days of school (min-max: 1–365), and caregivers lost 25 days of work (min-max: 0–365; Table 3). Victim and caregiver work losses translated to an average value of 106 714 RWF (112 USD) per family affected by a single snakebite, not including the value of lost education. Altogether, the average family incurred a total of 111 814 RWF (average: 117 USD; min-max: 0–1 535 USD) in direct and indirect losses per snakebite, not including long-term lost productivity or measures taken to prevent future bites. These estimates also excluded any costs associated with snakebite among domestic animals. Among the 479 families who owned livestock at the time of interview, six reported livestock that had been bitten in 2020 (1.3%). This figure rose to 6.2% when we asked families if their livestock had ever been bitten by a snake.

**Table 3:** Participant income and indirect losses reported by victims bitten by a snake in 2020 in Eastern Province, Rwanda.

	Mean	Min—Max
Household annual income^1^ (n = 629)		
RWF	219 259	0–5 760 000
USD	229	0–6 019
Participant annual income^1^ (n = 541)		
RWF	164 155	0–3 600 000
USD	172	0–3 762
Adult patient lost work due to snakebite (n = 523)		
# Days	41	0–365
RWF	80 057	0–727 445
USD	84	0–760
Caregiver lost work due to snakebite (n = 641)		
# Days	25	1–365
RWF	49 082	1 993–727 445
USD	51	2–760
**Total mean indirect household costs/case** ^**2**^		
# Days	54	0–730
RWF	106 714	0–1 454 890
USD	112	0–1 520

^1^Income included cash and the estimated value of goods produced e.g., crops but excluded income generated by anyone <18 years of age.

^2^Total costs include both patient and caregiver indirect costs.

## Discussion

Our study of snakebite victims in Eastern Province, Rwanda is unique among SSA assessments in that it evaluated patient costs incurred from both traditional healers and formal medical facilities. Overall, our evaluation confirmed the mental, physical, and financial burden that Rwandese households experience when people are bitten by snakes [19]. The typical adult victim lost at least 12% of the average annual Rwandese income in medical and productivity costs due to a single snakebite. Like other regions, snakebite victims in Rwanda were among the poorest of the poor, earning on average only 30% of the GNI [3,17]. Most victims were below the age of 50 years with nearly 25% under the age of 18 years. Approximately half reported long-term productivity losses, demonstrating a palpable societal blow to minors and working-age individuals. Moreover, snakebite survivors relied on personal savings, loans, and donations to pay treatment costs, suggesting a negative financial impact on entire communities. Altogether, Rwandese snakebite victims lose 7.4 million USD in out-of-pocket expenditures related to acute medical care and lost work. This illustrates a concerning picture of neglect for SBE care and recovery, with poverty as both a risk factor and a consequence of snakebite.

In Rwanda, families with no assets who are affected by permanent disability are eligible for free health insurance [20]. These individuals incur no medical expenses when presenting for treatment at government health facilities, in line with a government goal of elevating vulnerable families from extreme poverty. Moreover, patients from other ubudehe categories holding community-based health insurance should never pay more than 10% of their hospital bill when seeking government services [21]. Within this cohort, 115 respondents were insured through the low-income insurance program; however, 92 (80.0%) chose to access traditional medicine rather than receive free treatment at a health post or hospital. The remaining participants also preferred to access traditional medicine, regardless of insurance status. Rwandese snakebite victims seek traditional medicine due to convenience, advice from friends/family, and cultural beliefs [11]. The services provided commonly include herbal drinks/salves, wound cutting or burning, sucking venom, tourniquet, and black stone. Traditional healers are believed to charge less than medical doctors, to be able to protect against future bites, and to allow non-cash payments such as labor or agricultural goods. Many in Rwanda believe that physicians are not taught to treat snakebite [11]. There is also a likely financial reason for preferring traditional healers. Among those seeking hospital care, non-medical items (i.e., transport, food, accommodation) made up 65% of expenditures, representing a major barrier for low-income families to access hospital care, even when medical expenses were covered. Such costs are a known barrier for rural families in East Africa, who often need to travel long distances to access hospital care [22,23]. Moreover, delayed access to appropriate care (including antivenom) is a known risk factor for poor patient outcomes and death [24].

Snakebite survivors who sought care from hospitals in Rwanda lost approximately 32 121 RWF (34 USD) in direct medical and non-medical expenses. This estimate is far higher than the direct cost to treat patients suffering snakebite in Nigeria (12 USD), but lower than costs incurred by hospitals in Burkina Faso (49 USD) [7,25]. It is also lower than the average cost for pediatric poison control, including SBE treatment, in Morocco (157 USD) or the cost per patient incurred by hospitals in KwaZulu Natal (703–1 780 USD) [26,27]. Victims in Rwanda reported a longer mean hospital stay (6 days) versus Kenya (1–5 days), Nigeria (4.4 days) or Burkina Faso (2.6 days) [7,8,25]. Differences in costs and length of stay could be explained by supply shortages, as SAV is known to be a significant contributor to hospital costs (30.5% in Burkina Faso) and patient outcomes, but is not widely available among Rwandese health facilities [7,10,24]. It could also be related to care-seeking behaviour as respondents in this cohort often utilized traditional remedies prior to seeking hospital care. Use of traditional medicine following a snakebite has been associated with increased odds of death or disability elsewhere in Africa [25]. Although traditional medicine had a lower mean cost per patient than hospital care, the maximum cost was nearly five times higher than the maximum cost incurred at a hospital, demonstrating that the formal medical system can better protect victims against catastrophic financial losses. Lastly, economic evaluations of SBE in SSA are often from the hospital perspective and lack the details necessary to make direct comparisons.

In line with the World Health Organization NTD roadmap, the Government of Rwanda has ambitious goals related to SBE (50% reduction of morbidity and mortality by 2024) and economic growth (middle-income status by 2035) [28,29]. The high treatment and productivity costs described in this study suggest that increased investment in SBE prevention could be worthwhile, especially since the government managed insurance program absorbs the majority of costs for those accessing formal medical services. Moreover, development plans in Rwanda relying on significantly increased economic growth in the agricultural sector [30] are compromised by conditions limiting agricultural productivity, such as snakebite. Snakebite prevention in Rwanda could include community sensitization programs encouraging high risk populations to use bed nets, patch holes where snakes could enter homes, practice rodent control, use torches when travelling at night, wear closed-toed shoes, and to seek formal care immediately when bitten by a snake [4]. At the systems level, improved procurement and distribution of appropriate SAV among Rwandese health facilities in addition to the inclusion of SBE management in medical curricula, are likely to meaningfully improve patient outcomes [10,16,31]. Easing the burden of transportation could encourage more victims to seek immediate care from formal health facilities. Finally, public health agencies should consider formal engagement with traditional healers, given their frequent role as first responders to snakebite victims.

Despite best efforts, the economic losses presented in this paper should be considered minimum estimates. This is because our study was limited to snakebite survivors and because the total losses excluded medical costs associated with long-term physical or mental sequelae, productivity costs associated with long-term productivity decreases, and the value of lost education. This study did not address the direct costs co-paid by government health insurance plans. At least 32% of respondents felt they had not fully recovered from their bite, and this could contribute to long-term income loss and medical costs not included in this analysis. This study also excluded adolescent earnings and the cost of livestock who died, suffered reduced productivity, or required veterinary treatment due to SBE. Moreover, the concept of recovery appeared to be highly subjective as many participants who self-identified as fully recovered also reported ongoing physical limitations. Our participants were generally farmers and struggled to quantify personal and household salaries, which varied according to season and crops. To minimize recall bias, our enumerators used a series of probes to help respondents translate their harvests into cash estimates. If an individual provided conflicting or unsure estimates, their responses were excluded from analysis. Lastly, our study design aimed to provide provincial rather than national estimates of snakebite costs.

## Conclusions

In Rwanda, snakebite continues to cause serious hardship and economic losses, especially among the working poor. The total sum spent by snakebite victims on unproven traditional medicines is far higher than that spent on hospital care. Patients often incur debt to cover the costs of treatment as well as reduced income. These financial realities impede progress on national goals to eliminate poverty and to control NTDs. Investments are needed to engage traditional healers in snakebite management, to promote community-based health insurance schemes, to encourage prompt use of formal medical facilities, and to educate vulnerable populations on prevention methods.
